# The growth and cell population kinetics of spontaneous tumours in domestic animals.

**DOI:** 10.1038/bjc.1969.62

**Published:** 1969-09

**Authors:** L. N. Owen, G. G. Steel

## Abstract

**Images:**


### THE GROWTH AND CELL POPULATION KINETICS OF SPONTANEOUS TUMOURS IN DOMESTIC ANIMALS

L. N. OWEN AND G. G. STEEL

*From the Department of Animal Pathology, School of Veterinary Medicine, Madingley Road, Cambridge, and the Department of Biophysics, Institute of Cancer Research, Clifton Avenue, Belmont, Sutton, Surrey*

Received for publication April 5, 1969

INVESTIGATIONS into the relationship between cell proliferation and overall tumour growth rate in human cancer have to contend with the major limitation that only rarely can both cell kinetic data and information on volume growth rate be obtained in one and the same patient. When a case is operable it is usually inadvisable to delay treatment in order to estimate growth rate; when, for instance in the case of lung secondaries, it is right to leave tumours untreated, tissue specimens are seldom available.

It was to overcome this limitation that the present investigations were begun. In many cases of malignant disease in domestic animals the natural history and histopathological features are very similar to human cancer and in a proportion of these cases it is permissible to observe the course of the disease and perform simple investigations before treatment or euthanasia. The present communication covers the first nine cases which have been studied in this project.

The available data on the overall growth rate of untreated tumours in man have been reviewed by Steel and Lamerton (1966). The cases from which these data have been obtained form a highly selected group and one must be cautious in taking them to be typical of the majority of tumours, especially tumours in other anatomical sites. Some idea of the distribution of human tumour volume doubling times can be gained from the histogram shown in Fig. 1. This histogram shows the spread of 175 measurements of human tumour growth rate; 135 of these were metastatic deposits in the lung (Collins, 1962; Collins, Loeffler and Tivey, 1956; Breur, 1966); 34 were primary lung tumours; 6 were primary bone tumours (Spratt, 1965). The mean values for these three classes of tumour are not significantly different. The median volume doubling time of the whole group is 66 days and 90% of the values fall in the range 16–253 days.

Cell proliferation studies by the method of labelled mitoses have at the present time only been reported in two series of cases of human cancer. Clarkson *et al.* (1965) has described detailed kinetic studies on neoplastic effusions in six cases. Frindel *et al.* (1968) have reported their investigations on five superficial solid tumours. Many more cases are recorded in which a thymidine labelling index has been determined, either by *in vivo* or by *in vitro* thymidine administration. These have been summarised by Steel and Lamerton (1966) and compared with the available data on human tumour growth rate. The conclusion from this work was that in the majority of human tumours, cell loss could be the dominant factor determining growth rate. The same conclusion has been reached by Iversen (1967) and Refsum and Berdal (1967) on the basis of measurements of mitotic rate in tumours of the head and neck by the colchicine method; their calculations implied that the rate of cell loss must in their sample of tumours be in excess of 90% of the rate of cell production.

FIG. 1.—The distribution of volume doubling times for tumours in man and in domestic animals. The human data are collected from the literature (Steel, 1967). The animal data are from the present study: the numbers indicate the case number of individual lung metastases or primary tumours; for cases 6 and 9 the range of doubling times is shown.

The main source of uncertainty in both of these estimates of cell loss from human tumours probably lies in the fact that the growth rate and cell kinetic data had to be taken from different series of cases. The object of the present studies on spontaneous tumours in domestic animals was to study thymidine labelling in tumours of known growth rate and also to gather data on the overall growth rate of tumours in species which are intermediate in size and longevity between man and laboratory animals.

### MEASUREMENTS OF TUMOUR GROWTH RATE

### *Access to dogs*

Dogs with malignant neoplasms have been admitted to the Cambridge University Veterinary Hospital for surgical or radiotherapeutic treatment. When lung metastases developed the general health of the animal was usually not immediately affected and in some cases it has been possible to take serial radiographs of the chest before euthanasia became necessary.

### *Details of X-ray procedure*

Radiographs were made on a machine with a tube distance of 75 cm. In most instances a Potter–Buckey diaphragm was used and for the larger dogs a Schoenander grid was found to be an advantage. A standard arrangement was used for each dog.

In cases 1 and 2, forced inspiration was used to improve the radiographic demonstration of metastases. Dogs were premedicated with atropine sulphate 0·65 mg. and acetyl-promazine 0·5 to 3 mg. intramuscularly. Anaesthesia was induced with methohexitone sodium (Brietal) and maintained with nitrous oxide and oxygen, deepened with halothane if required. A semi-open to and fro Magill tube was used, the expiratory valve being completely or partially closed, and the rebreathing bag compressed manually. After chest radiography the dog was allowed to recover and was usually able to walk in 10 to 15 minutes. The technique could thus be used successfully on an outpatient basis.

Radiographs in a lateral and postero-anterior plane were taken but the lateral radiographs proved to be more valuable in this study.

*Method of measuring metastases on radiographs*

Simple Vernier callipers were used to measure the pulmonary metastases as outlined on the radiographs. Most of the metastases measured were round but in a few cases one diameter was slightly larger than the other. The use of a transparent plastic rule on which circles of increasing diameter were engraved was found to be an advantage (Breur, 1966). Tumour volume was computed as $\pi/6$ times the cube of the mean diameter. Growth curves (Fig. 2) were plotted on semi-logarithmic paper and the doubling time recorded in Table I was that defined by the last few experimental points.

TABLE I

| Case | Tumour | Volume doubling time (days) | Labelling index (%) | Potential doubling time (days) | Cell loss factor (%) | Cell loss factor Grade |
|---|---|---|---|---|---|---|
| 1 | Secondaries (1) | 22 | 3·3 | 7·6 | 66 | III |
| | (2) | (6·8) | 6·6 | 3·8 | 44 | II |
| | (3) | 6·8 | 5·3 | 4·7 | 31 | II |
| | (4) | 6·8 | 3·4 | 7·4 | 0 | I |
| 2 | Primary | 80 | 5·0 | 5·0 | 94 | IV |
| | Secondaries (1) | 150 | 4·6 | 5·5 | 96 | IV |
| | (2) | 60 | 6·2 | 4·0 | 93 | IV |
| 3 | Secondaries (1) | 17 | 6·9 | 3·6 | 79 | III |
| | (2) | 15 | 7·9 | 3·2 | 79 | III |
| | (3) | 12 | 8·3 | 3·0 | 75 | III |
| 4 | Primary | 38 | 6·9 | 3·6 | 90 | IV |
| 6 | Secondaries | 10 | 10·8 | 2·3 | 77 | III |
| | | 20 | 9·0 | 2·8 | 86 | III |
| | | (range 7–25 days) | | | | |
| 7 | Secondaries (1) | 16 | 3·6 | 7·0 | 56 | III |
| | (2) | 16 | 1·4 | 14 | 12 | I |
| | (3) | (16) | 1·6 | 16 | 0 | I |
| | (4) | 18 | 2·6 | 9·6 | 47 | II |
| 8 | Primary | (25) | | | | |
| | Secondaries (1) | 40 | < 1·0 (Secondaries 1–4) | | | I |
| | (2) | (40) | | | | |
| | (3) | 30 | | | | |
| | (4) | 40 | | | | |
| 9 | 7 Secondaries | mean of 24 days | 9·7 | 2·6 | 89 | III |

FIG. 2.—Growth curves of lung metastases (solid lines) and primary tumours (broken lines) for tumours in domestic animals.

*Case* 1

Terrier. Male. Age 16 months. Weight 6·4 kg. Thymidine dose 2 mCi. A fibro-sarcoma in the right lumbar region, not involving bone, was excised two months after it was first noticed. There was obvious recurrence of this primary tumour within 3 weeks of surgery and shortly after this metastases were visible radiographically in the lungs.

Measurements of tumour volume were made on six occasions over a period of 41 days. The secondary tumour numbered (1) (Table I) was visible throughout this period and had a doubling time of 22 days. Secondaries (3) and (4) were not seen on the earlier radiographs; on the later ones both had doubling times of 6·8 days. The three measurements on secondary (2) do not allow a reliable doubling time to be estimated but they are consistent with the figure of 6·8 days.

This case is an exception to the generalisation that multiple lung secondaries usually have comparable growth rates (Breur, 1966; Smithers, 1968). The metastasis which was radiographically visible first was, at the time of death, the smallest of the four which were measurable.

*Case* 2

Alsation. Male. Age 9 years. Weight 30 kg. Thymidine dose 6 mCi. When seen, the animal had been lame 3 months on the right fore limb. A diagnosis of sclerotic osteosarcoma of the right radius and ulna was made. Therapy was attempted using an intravenous injection of tritiated " Synkavit ". It was appreciated that the residual tritium from this injection might confuse the tritiated thymidine autoradiographs. In fact, after the delay of 18 weeks, the only evidence of the $H^3$-Synkavit was diffuse labelling in and around necrotic regions. It seemed justifiable to measure the thymidine labelling index by counting those cells which showed only nuclear labelling.

The growth curve for the primary tumour showed a negligible response to Synkavit therapy. Up to 70 days after therapy the doubling time was about 40 days, though the final measurement suggests that this may have more than doubled by the time of death.

Secondary (1) was clearly reduced in size as a result of therapy. It soon regrew, however, and finally had a doubling time of 150 days. Secondary (2) was not visible before therapy. Its growth rate gradually decreased and the terminal doubling time was about 60 days.

*Case* 3

Retriever. Male. Age 5 years. Weight 31 kg. Thymidine dose 6 mCi. A spindle-cell sarcoma of the soft tissues of the right carpal region was diagnosed and given 1200 R of X-irradiation. The tumour regressed but recurred 8 months later when it was irradiated with 1000 R. Metastases in the lungs were present at this time.

Chest radiographs were taken on six occasions in the period of 5 weeks before death. Three secondaries could be measured throughout this period. The growth curves were not exponential and there is some uncertainty in the estimates of terminal growth rate, but it seems possible that secondary (3) had a shorter doubling time (about 12 days) than secondaries (1) and (2) (about 16 days).

*Case* 4

Ginger neutered male cat. Age 13 years. Weight 3 kg. Thymidine dose 0·6 mCi. A non-painful hard irregular swelling on the left 6th rib was excised but recurred 5 weeks later. Histological examination showed the tumour to be an osteosarcoma.

The measurements of the primary tumour were made partly by vernier calipers (width measurement) and partly from radiographs (length and depth). The depth measurement was particularly uncertain. The estimate of 38 days for the volume doubling time is therefore probably less precise than for many of the lung secondaries in the present series of cases.

*Case* 5

Cross-bred Scottish terrier. Bitch. Age 10 years. Weight 18 kg. Thymidine dose 3·5 mCi. This animal had a diffuse anaplastic mammary carcinoma with gross infiltration of the skin. The tumour had been known to be present at least 2 months.

No estimate of the rate of growth was possible in this case.

*Case* 6

Blue roan Cocker Spaniel. Male, Age 11 years. Weight 10 kg. Thymidine dose 4 mCi. An ulcerated melanoma on the gum adjacent to the left mandibular canine was excised but recurred within 2 months. At the time of recurrence, radiographic examination showed lung metastases.

The measurements of growth rate in case 6 were the most detailed of all the cases in the present series. It was possible to obtain growth curves on 11 lung secondaries, varying over a total size range of 0·03 c.c. to 6·4 c.c. These presented a range of terminal volume doubling times, but it was noticeable that seven formed a relatively uniform group (12·4, 12·5, 13·4, 14·0, 14·6, 15·0, 15·5 days) while two were considerably faster than the main group (7·0 and 7·5 days) and two were slower (22·6 and 24·0 days). It then became clear that there was also a size difference between these three groups (their mean growth curves are shown in Fig. 2): the main group had a mean terminal volume of 2·53 c.c., the faster group 1·37 c.c. and the slower group 5·6 c.c. The implication is that within the whole group of lung secondaries in this animal, size and volume doubling time were positively correlated and furthermore that all the secondaries might well be following the same non-exponential growth curve. The situation is that which would be expected if the various metastases were initiated at different times but followed the same growth curve, some being found earlier in their growth than others. It is possible to reproduce a portion of such a growth curve by translating the individual curves in time until the points fall on a smooth curve. The curve obtained by this procedure is shown in Fig. 3. It has a doubling time of 7 days at first, increasing to 25 days for the larger tumours, as would be expected from the individual doubling time values quoted above.

As can be seen from the photograph of the post-mortem specimen (Fig. 4) the secondaries from this tumour were highly pigmented. This to some extent made the counting of the autoradiographs difficult. Although under the microscope the black silver grains usually stood out well against the brown of the melanin pigment, the latter was sometimes so intense as to obscure cell nuclei (Fig. 5C).

FIG. 3.—Combined growth curve for 11 lung metastases in case 6 (see text).

Repeat autoradiographs were therefore made after bleaching out the melanin with 0·05% potassium permanganate followed by 0·3% oxalic acid.

*Case* 7

Border Collie. Bitch. Age 11 years. Weight 17 kg. Thymidine dose 4 mCi. An adenocarcinoma of the fourth left mammary gland was diagnosed. Metastases were present in the left os calcis, right femur and the caudal vertebrae as well as in the lungs.

Of the four lung metastases which were measurable, one (number 4) was visible over a total period of 28 days and had a well-defined doubling time of 18 days. The other three were measurable for a shorter period, were smaller and may have had a slightly shorter doubling time of 16 days. Only two measurements were possible on metastasis number (3) and these were consistent with the growth rate of the other secondaries.

*Case* 8

Miniature Poodle. Female. Age 7 years. Weight 9 kg. Thymidine dose 2 mCi. The animal presented with a tumour in the right inguinal mammary gland and with metastases in the lungs. Histological diagnosis was spindle cell sarcoma.

The four metastases whose growth could be followed differed widely in size but they all had volume doubling times of about 40 days.

The autoradiographs from this case presented severe counting problems. The metastases consisted of large areas of virtually unlabelled acellular tissue with a few well-labelled islands. Because of the difficulty of making counts which were representative of the whole tumour, only an upper limit on the labelling index is given in this case (Table I). It nevertheless seemed that the labelling index in case 8 was lower than for the other tumours of this series.

*Case* 9

Crossbred Alsatian. Male. Age 6 years. Weight 29 kg. Thymidine dose 6 mCi. This animal presented with a firm irregular swelling around the upper third of the right radius and ulna. Radiographs indicated an osteolytic bone tumour with numerous lung secondaries, and on biopsy of the primary, a diagnosis of osteosarcoma was made (a very unusual site). Chest radiographs were made over a period of 46 days, and it was possible to follow the growth of 7 metastases. Growth curves for 4 of these are shown in Fig. 2. The terminal volume doubling times were estimated to be 11, 25, 40, 26, 20, 37 and 12 days (mean = 24 days). Biopsies were obtained of metastatic lung tumours of a similar size to those which had been measured radiographically.

### STUDIES OF THYMIDINE LABELLING

Tritium-labelled thymidine (TRK.61, Radiochemical Centre, Amersham, England) was injected intravenously at a specific activity which ranged between 15 and 25 curies/mM. After an interval of 1-2 hours, the animals were destroyed with pentobarbitone sodium and a thorough post-mortem examination was performed. Representative specimens of primary and secondary tumours were fixed in 10% neutral formol saline. In cases where there were many lung metastases great care had to be taken to identify those metastases which had been measured. The identification of specific metastases was facilitated by inflating the lungs and comparing the anatomical location of the metastases with their position as shown on radiographs taken immediately before death.

Paraffin sections were cut at 4 $\mu$ and autoradiographed by the dipping technique using Ilford K5 emulsion (Lord, 1963). Some slides were prestained by the Feulgen reaction, others were stained with haematoxylin and eosin after photographic processing. Test slides were examined after various exposure times; good labelling was usually found after 1 to 3 months exposure, though some slides had to be left for up to 6 months (Fig. 5).

Many of the tumours showed extensive macroscopic and microscopic necrosis and the degree of heterogeneity made it difficult to be sure that counts of labelled cells were representative of the whole tumour. The standard counting procedure was as follows. Using an overall magnification of 400× or 1000× (oil immersion objective), counts were made within at least 40 fields defined by an eyepiece graticule which gave a square field with 16 subdivisions. Fields were selected with the aid of vernier scales on the microscope stage, giving an array which covered the specimen in a 1 mm. grid. Within each field the number of

---

EXPLANATION OF PLATES

FIG. 4.—Post-mortem appearance of lungs from case 6.

FIG. 5.—Examples of the autoradiographs from: (A) lung metastases in case 1; (B) intestinal epithelium in case 1; (C) case 6, showing labelling within a region of heavy pigmentation; (D) case 1, showing the wide range of grain counts over individual nuclei; (E) labelled cell and unlabelled mitotic figure (arrowed) in case 5; (F) labelled cells and one labelled mitosis in case 9.

FIG. 9.—Photomicrographs of the autoradiographs from case 3 showing (A) labelling in a lung metastasis; (B) labelling in adrenal cortex; (C) labelling in capsule of the kidney; (D) mitotic figures but no labelling in kidney metastasis. (B), (C) and (D) are from different parts of the same microscopic slide.

4

Owen and Steel.

Owen and Steel.

9

Owen and Steel.

subdividing squares occupied by viable tissue, the number of labelled cells and the number of mitotic figures were recorded. In a representative proportion of the fields an estimate was also made of the total number of nuclei per full microscopic field; this was the most difficult parameter to measure reliably. From these data it was possible to calculate the labelling and mitotic indices. The labelling index results (labelled cells per 100 cells) are given in Table I.

*Cell cycle investigation by the method of labelled mitoses: cases* 5 *and* 9

Case 5 was selected because the large area of tumour tissue infiltrating skin made practicable the removal of repeated biopsies. Fifteen of these were taken, first at about 4-hour intervals and later at about 8-hour intervals up to 3 days after thymidine injection. The specimens were fairly small and in the autoradiographs (3 months exposure) the total number of mitoses found was in the range 20–50 per specimen. There is a correspondingly large statistical counting error.

The resulting plot (Fig. 6) of labelled mitoses as a fraction of all mitoses gives information on the duration of the cell cycle and its constituent phases (Quastler

Fig. 6.—Labelled mitoses curves from case 5 (top) and case 9 (bottom). The full lines are the computed best-fitting theoretical curves (see text). For case 9 the circles indicate counts on lung metastases, the triangles on biopsies of the primary tumour.

and Sherman, 1959; Mendelsohn, Dohan and Moore, 1960; Steel, Adams and Barrett, 1966). By comparison with other published labelled mitoses curves in tumours, that for case 5 shows heavy damping and the first peak is rather poorly defined. Both of these characteristics are consistent with considerable spread in the durations of the various phases of the cell cycle. The data have been analysed by the method of Barrett (1966). An optimising computer program has been used (Steel and Hanes, unpublished) in which the means and standard deviations of $G_2$, S and $G_1$ are varied until a curve is found which has the minimum mean square deviation from the experimental points. The resulting curve is shown in Fig. 6. It corresponds to the following values of the phase durations (mean $\pm$ standard deviation in hours; mitotic time is assumed to be 1 hour and to be included in the means of $G_1$ and $G_2$).

$$G_2 = 5{\cdot}4 \pm 1{\cdot}5$$

$$S = 5{\cdot}3 \pm 1{\cdot}7$$

$$G_1 = 61 \pm 48$$

The analysis indicates a median cycle time of 50 hours, though in view of the quality of the data this value is subject to considerable uncertainty.

The above data must be accepted with some caution. Some of the characteristics of the labelled mitoses curve of Fig. 6 could also have been produced by incomplete thymidine labelling. The ratio of the count of labelled nuclei to mitotic figures was extremely low (about 2) and this might well imply that not all DNA-synthesising cells were seen to be labelled in the autoradiographs. The fact that the first peak of the labelled mitoses curve reached 88% implies that of the cells which were in the middle of their DNA synthetic period at the time of thymidine injection, less than about 10% were missed; however, labelled cells could more easily be missed if they were labelled when at the beginning or end of the DNA synthetic period since they would probably have a smaller uptake of thymidine. A further explanation of the low ratio of labelled cells to mitoses could be a relatively long duration of mitosis. The fact that the first limb of the labelled mitoses curve rises rather slowly is consistent with this.

The autoradiographs from this case have been repeated with no improvement in the quality of definition of the curve. Longer exposures than 3 months have not been used because by this time some nuclei were so heavily labelled as to prevent the reliable identification of mitosis.

The main conclusion which can be drawn from the data on this case is that although there are some peculiar features about the labelled mitoses curve, which may have resulted from great variations in the availability of tritiated thymidine to individual cells, the duration of DNA synthesis was not more than about 8 hours. The duration of DNA synthesis in this tumour was thus comparable to values for most tumours in experimental animals (Frindel *et al.*, 1967; Mendelsohn *et al.*, 1960; Steel *et al.*, 1966) and only perhaps half as long as has been found in some human tumours (Frindel, Malaise and Tubiana, 1968).

In case 9, an attempt was made to perform a labelled mitoses experiment actually on lung metastases. This is clearly the ideal experiment since the rest of the data on growth and labelling index are on lung secondaries; to the knowledge of the authors this has not, however, previously been attempted with spontaneous lung metastases.

The animal was first anaesthetised with pentobarbitone sodium given intravenously, and placed on a ventilator. 6 mCi of tritiated thymidine was injected intravenously. A thoracotomy was performed on the right side and the brachial and brachiocephalic arteries clamped. Pupils dilated after 5 minutes 20 seconds. The clamp was removed 3 minutes later and the pupils constricted. Following this brain anoxia no further anaesthetic was required for 12 hours. An intravenous drip of 4·3% dextrose and 0·18% sodium chloride was given to balance fluid loss. Individual lung metastases were removed and biopsies of the primary were made at intervals until death of the animal 20 hours after thymidine injection.

Autoradiographs prepared after 3 months exposure were of good quality and the identification of labelled and unlabelled mitotic figures could be made with confidence. In the lung metastases (Fig. 6) the first peak of the labelled mitoses curve was well defined and the optimum theoretical curve through the points has the parameters

$$G_2 = 6{\cdot}6 \pm 4{\cdot}1 \text{ hours}$$

$$S = 11{\cdot}1 \pm 2{\cdot}6$$

$$G_1 = \text{(not defined)}$$

The labelling index of the earliest biopsies of lung metastases was 9·7%. The counts on biopsies of the primary tumour suggest that the duration and spread of $G_2$ values were similar to those found for the metastases. However, the points at 16 and 20 hours show little evidence of a fall in the percentage of labelled mitoses. It must therefore be concluded that the duration of DNA synthesis in the primary tumour was in excess of 15 hours.

*Evidence for incomplete thymidine labelling: case* 3

Many of the tumours in the present series showed considerable heterogeneity. There were extensive regions of necrosis, regions of infiltration by mononuclear cells and wide variations in the number of nuclei per unit area of section. Morphological heterogeneity was often reflected in a very non-uniform distribution of labelled cells. In a few autoradiographs the clear impression was gained that thymidine labelling dose to blood vessels was high (both in labelling index and grain count) and that elsewhere it was lower; occasional vessels were found intersecting the plane of the section around which there was an apparent concentration of labelled cells. This observation corresponds to the situation which has been reported in spontaneous and transplanted tumours in the mouse (Kligerman, Heidenreich and Greene, 1962; Tannock, 1968) and which has largely been attributed to variations in the availability of metabolites at different distances from blood vessels, with consequent variations in the rate of cell proliferation.

An observation in case 3 which is disturbing from the point of view of thymidine technique, was that regions within the lung metastases could be found in which labelling was low or absent but in which mitotic figures were still clearly visible. An analysis was made of the spatial distribution of labelled cells and mitotic figures in the metastasis designated (3) and the results are shown in Fig. 7. Using the standard counting system mentioned earlier, counts of labelled cells and mitoses were made in array of fields each of size 200 × 200 microns. The distribution for mitotic figures has a variance equal to 1·26 times the mean and it is well fitted by a Poisson distribution with the same mean (5·75 mitoses/field). It would therefore seem that in this tumour the mitoses were distributed at random. When the same procedure was applied to the labelled cells a wide discrepancy was found; the experimental variance is 4·6 times the mean and the labelled cells were thus not randomly distributed. This may be emphasised by the type of plot shown in Fig. 8, where for each field the ratio of labelled cells to mitoses has been calculated

Fig. 7.—Spatial distribution of mitotic figures and labelled cells in case 3. The broken lines indicate Poisson distributions having the same mean as the experimental data.

and plotted against the number of labelled cells. There is a clear correlation between these two parameters, a correlation which should not exist if thymidine were reaching all proliferating cells. The only possible explanation apart from incomplete thymidine availability is that in areas of poor metabolite supply the duration of mitosis was increased and that mitoses may have been arrested. But it seems unlikely that this could occur to just the necessary extent to produce a random distribution of mitotic figures.

Examination of autoradiographs of the kidney also showed tumour cells which had failed to label. On the first batch of autoradiographs which were prepared, one metastasis was found just beneath the capsule. A detailed examination revealed clearly labelled cells within the capsule of the kidney (Fig. 9) but in the tumour tissue, which came to within 0·5 mm. of the site of these labelled cells, no labelling could be found. This was in spite of the fact that the kidney metastasis had a mitotic index of 0·61%, comparable with the mitotic indices of the three measured lung metastases (0·65%, 0·72%, 1·17%). A section of the adrenal gland on the same slide as the kidney metastases was heavily labelled.

FIG. 8.—Correlation between the number of labelled cells per mitotic figure and the number of labelled cells per microscope field in case 3.

The failure of cells within the kidney metastasis to label has been confirmed by repeated autoradiographs, with up to 6 months exposure. On re-examination of the post-mortem specimens, four further metastases were found in the kidney. Autoradiographs were made of these and it was found that one (5 mm. diameter) contained labelled cells while the other three (diameter 1 mm., 3 mm. and 12 mm.) were unlabelled. A single metastasis in the heart was well labelled.

The cause of the anomalous labelling in kidney was almost certainly vascular occlusion due to tumour emboli and to pathological changes with the kidney itself. The tubules showed necrosis and there was an interstitial nephritis, a common condition in older dogs. The fact that the kidney was abnormal does not, however, remove the problems posed by the failure to find labelling in mitotically active tumour tissue. If the blood supply to a region of the kidney was so poor as to completely prevent thymidine labelling, how could it be that the mitotic index was unaffected? If such a situation could arise in this pathological situation, could it not also arise in other cases of regional thrombosis in tumours? It is possible that the blood supply deteriorated suddenly, just before thymidine injection, but it would be a coincidence if this happened in time to prevent thymidine reaching the tumour but not so long beforehand that mitotic figures could disappear. The observation of labelled cells in the capsule is reasonable because of the separate blood supply which it receives.

The conclusion which can be drawn from the studies of thymidine labelling in case 3 is that in both the lung and the kidney metastases there is evidence for incomplete thymidine labelling. In both tissues, regions were found in which the mitotic index was normal but in which thymidine labelling was much reduced or absent. The question whether labelling could have been detected by extremely long exposures cannot be conclusively answered. In the kidney metastases this is unlikely, because after an autoradiographic exposure of 3 months not one cell was found to have more than 4 grains. Even if the non-uniformity of labelling in the lung metastases could be reduced or abolished by long exposures, it would still remain that the observation of high grain counts over some cells within a tumour cannot be taken to imply that labelling is everywhere complete. Among the dog tumours of this series there were other examples of what was apparently a wide distribution of grain counts; as was noted in the discussion of case 5 it was difficult to find an autoradiographic exposure which allowed recognition of the low-labelled cells without heavy labelling obscuring the appearance of other nuclei.

### DISCUSSION: COMBINATION OF THE RESULTS ON GROWTH RATE AND LABELLING INDEX

The measurements of volume doubling time and thymidine labelling index may be brought together in the type of chart shown in Fig. 10. For tumour cell populations from which there is no cell loss, the cell population doubling time should be related to the labelling index (L.I.) and duration of the DNA synthetic period ($t_S$) by the relation

$$T = \lambda \frac{t_S}{\text{L.I.}} \qquad \ldots \text{(i)}$$

When applied to tumour cell populations in which cell loss occurs, T has been called the potential doubling time (Steel, 1967). A theoretical discussion of the parameter $\lambda$ has been presented (Steel, 1968) where it is shown that although $\lambda$ has a possible range of values from 0·7 to 1·4, values higher than unity are unlikely, and when the population doubling time is longer than 2 days, $\lambda$ will be within the range 0·7 to 0·8. In practice, cell *population* doubling times can seldom be measured; usually it is assumed that a volume doubling time gives the same result. This may not be the case, but it should be noted that most of the factors which would upset the equivalence of population and volume doubling time, such as the progressive accumulation of necrotic material, will tend to make the doubling time of volume *shorter* than that of the cell population. With this qualification, equation (i) allows one to estimate the doubling time which a tumour should have in the absence of cell loss. Comparison of such a potential doubling time with the true volume doubling time of a tumour ($T_d$) enables one to deduce the extent of cell loss. If a cell loss factor is defined as the rate of cell loss as a fraction of the rate at which cells are being added to the tumour volume, then it can be shown that

$$\text{Cell loss factor} = 1 - \frac{T}{T_d} \qquad \ldots \text{(ii)}$$

(Steel, 1968)

In Fig. 10 the present measurements of volume doubling time and labelling index have been plotted together using double-logarithmic coordinates, and on such a plot the relationship of equation (i) gives a series of straight lines for various values of $t_S$. These may be called the no-loss relationships and it is to be expected that experimental points will fall to the right hand side of the appropriate no-loss line to an extent which increases with the cell loss factor. Points which are far to the right indicate high cell loss.

FIG. 10.—Correlation between labelling index and volume doubling time for tumours in domestic animals. The circles enclosing the case number represent individual lung metastases; the stars represent primary tumours. The full line indicates the no-loss condition for an assumed S period of 8 hours and the broken lines correspond to cell loss factors of 50% or 90%.

In the present calculations a value of $\lambda$ of 0·75 has been used and $t_S$ has been taken to be 8 hours, on the basis of cases 5 and 9. There is some uncertainty in this figure but the choice of 8 hours is to some extent supported by the available data on tumours in experimental animals and in man (Lamerton and Steel, 1968). It can be seen that the results indicate extensive cell loss, particularly in the metastases of case 2 and in both of the primary tumours. Values of cell loss factor have been calculated and are given in Table I. It should be emphasised that the possible error in the calculations of cell loss factor may be considerable. Apart from the measurement errors of the labelling index and volume doubling time, the assumptions that $t_S$ is known and constant and that the volume doubling time reflects the cell population doubling time introduce considerable uncertainty. The most rational course may be to broadly grade tumours on the basis of cell loss factor. This has been done in Table I where the grades are defined as follows:

| | Cell loss factor | Ratio of potential to observed doubling time |
|---|---|---|
| Grade I | less than 20% | greater than 8/10 |
| Grade II | 20% to 50% | 8/10 to 1/2 |
| Grade III | 50% to 90% | 1/2 to 1/10 |
| Grade IV | greater than 90% | less than 1/10 |

The evidence for incomplete thymidine labelling which was particularly clear in case 3 is a cause for some concern to those who use tritium labelled thymidine in the study of tumour growth. It appears that in some tumours there is a very broad distribution of grain counts following a single thymidine injection, and that it is necessary to expose the autoradiographs until some cells are very heavily labelled in order to detect a substantial proportion of weakly labelled cells. In a labelled mitoses experiment this cannot be done without biasing the choice of mitotic figures. The impression has been gained that this problem is more serious in canine tumours than in tumours of the rat and mouse, but no satisfactory comparison of this type has so far been made. It should be noted that if the labelling indices recorded in Table I are distorted by incomplete thymidine labelling then the estimates of cell loss factor deduced from them will be too low rather than too high: the necessity to postulate cell loss arises because *too many* labelled cells are seen to explain the observed volume growth rate.

An assumption which is inherent in the present calculations of cell loss factor is that cells incorporate tritiated thymidine only as a preparation for division. It is thus assumed that the type of labelling which has been observed by Pelc (1964) does not occur extensively in tumours. What evidence there is goes against this possibility: in case 3 the use of mitotic index rather than labelling index would *increase* the estimate of cell loss factor, and it should be remembered that the work of Iversen (1967) and Refsum and Berdal (1967) indicated high cell loss by the colchicine method.

As regards the comparative aspects of tumour growth rate it is difficult on such a small number of cases to draw any firm conclusions. In Fig. 1 the volume doubling times for the present series have been represented on the same scale as the available human data. If the doubling times of the metastases in each case are first averaged to give a single value, then the median doubling time for the primary and secondary animal tumours is about 25 days, somewhat shorter than the median of 66 days for the human tumours. The difference between the distributions of doubling time for tumours in domestic animals and man may be significant ($P = 0{\cdot}05$, calculated from the distributions of the logarithm of doubling time). A comparison of labelling indices is also consistent with a slightly higher growth rate in the animal tumours: the median labelling index for the tumours in the present series is about 5%, as compared with a median of 3% for a group of 170 human tumours (Steel, 1967). While these differences are in the direction which one might expect from the differences in lifespan they are nevertheless sufficiently small to confirm the basic starting point of this work that spontaneous tumours in domestic animals serve as a good model for human cancer. It can be concluded, therefore, that the tentative hypothesis that in a majority of human tumours cell loss is the dominant factor determining growth rate has been to some extent confirmed by the present simultaneous studies of labelling index and growth rate in spontaneous animal tumours.

SUMMARY

The growth rate of spontaneously arising tumours has been studied in seven dogs of various breeds and in one cat. In most cases the measurements were made on multiple lung metastases, though where possible the growth of the primary tumour was also followed. The volume doubling times ranged from 7 days to 150 days, on average slightly shorter than the published values of human tumour doubling times.

Autoradiographic studies of the uptake of tritium-labelled thymidine were made in all cases. In two cases the technique of labelled mitoses was used: in one dog, biopsies of infiltrated skin were taken; another dog was kept anaesthetised throughout the experiment and lung metastases and biopsies of the primary tumour were removed at intervals up to 20 hours. There was evidence that in some canine tumours $H^3$-thymidine did not reach all cells engaged in DNA synthesis in detectable quantities. In spite of this, calculations of the rate of cell production based on the measured thymidine labelling indices were much higher than was necessary to maintain the observed growth rates. The results therefore imply that extensive cell loss was taking place from the tumours of domestic animals. On rather less satisfactory evidence this has previously been suspected to be the case in a majority of tumours in man.

We are grateful to Mrs. J. Lucas for her skill in the preparation of the autoradiographs and to Mr. H. D. Williamson for the radiography of the lung metastases.

### REFERENCES

Barrett, J. C.—(1966) *J. natn. Cancer Inst.*, **37**, 443.
Breur, K.—(1966) *Eur. J. Cancer*, **2**, 157.
Clarkson, B., Ota, K., Ohkita, T. and O'Connor, A.—(1965) *Cancer, N.Y.*, **18**, 1189.
Collins, V. P.—(1962) *Cancer, N.Y.*, **15**, 387.
Collins, V. P., Loeffler, R. K. and Tivey, H.—(1956) *Am. J. Roentg.*, **76**, 988.
Frindel, E., Malaise, E. P., Alpen, E. and Tubiana, M.—(1967) *Cancer Res.*, **27**, 1122.
Frindel, E., Malaise, E. and Tubiana, M.—(1968) *Cancer, N.Y.*, **22**, 13.
Iversen, O. H.—(1967) *Eur. J. Cancer*, **3**, 389.
Kligerman, M. M., Heidenreich, W. F. and Greene, S.—(1962) *Nature, Lond.*, **196**, 282.
Lamerton, L. F. and Steel, G. G.—(1968) in ' Progress in Biophysics and Molecular Biology '. Edited by Butler, J. A. V. and Noble, D. London (Pergamon Press).
Lord, B. I.—(1963) *J. photogr. Sci.* **11**, 342.
Mendelsohn, M. L., Dohan, F. C. and Moore, H. A.—(1960) *J. natn. Cancer Inst.*, **25**, 477.
Pelc, S. R.—(1964) *J. Cell Biol.*, **22**, 21.
Quastler, H. and Sherman, F. G.—(1959) *Expl Cell Res.*, **17**, 420.
Refsum, S. B. and Berdal, P.—(1967) *Eur. J. Cancer*, **3**, 235.
Smithers, D. W.—(1968) *Clin. Radiol.*, **19**, 113.
Spratt, J. S.—(1965) *Cancer, N.Y.*, **18**, 14.
Steel, G. G. and Lamerton, L. F.—(1966) *Br. J. Cancer*, **20**, 74.
Steel, G. G., Adams, K. and Barrett, J. C.—(1966) *Br. J. Cancer*, **20**, 784.
Steel, G. G.—(1967) *Eur. J. Cancer*, **3**, 381.
Steel, G. G.—(1968) *Cell and Tissue Kinetics*, **1**, 193.
Tannock, I. F.—(1968) *Br. J. Cancer*, **22**, 258.


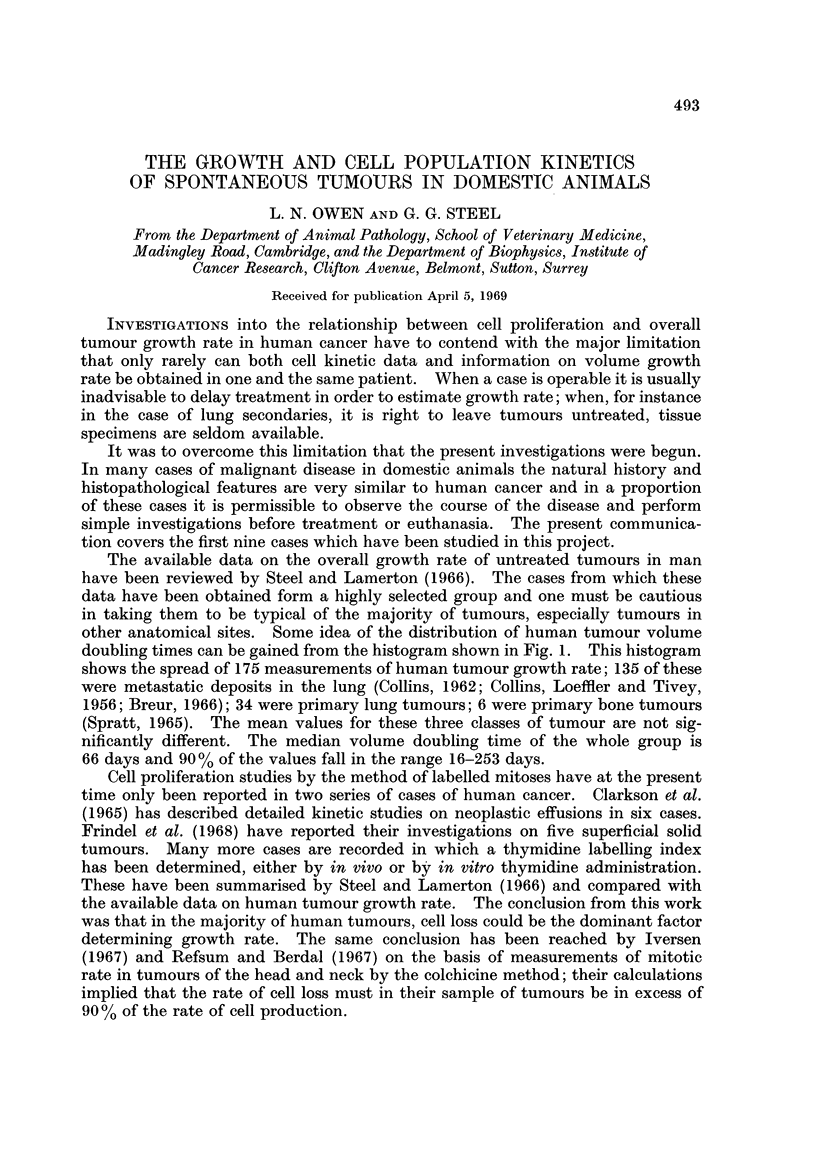

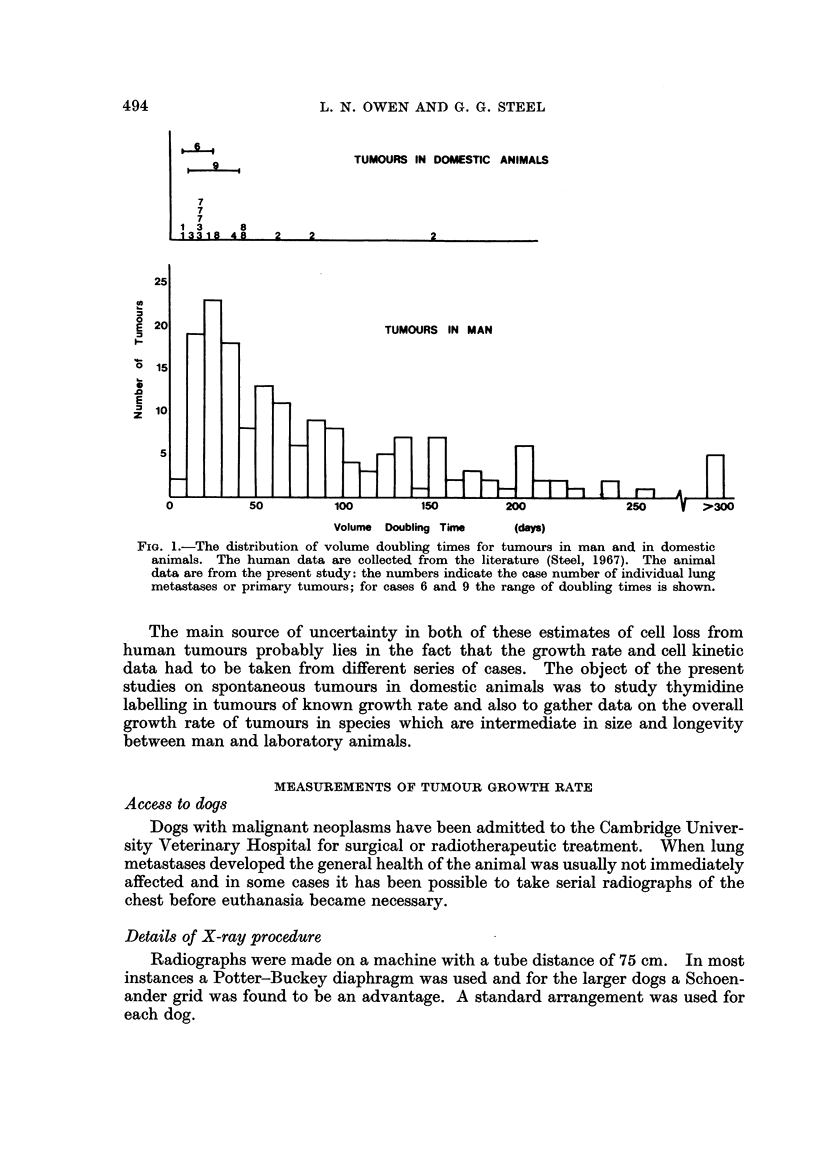

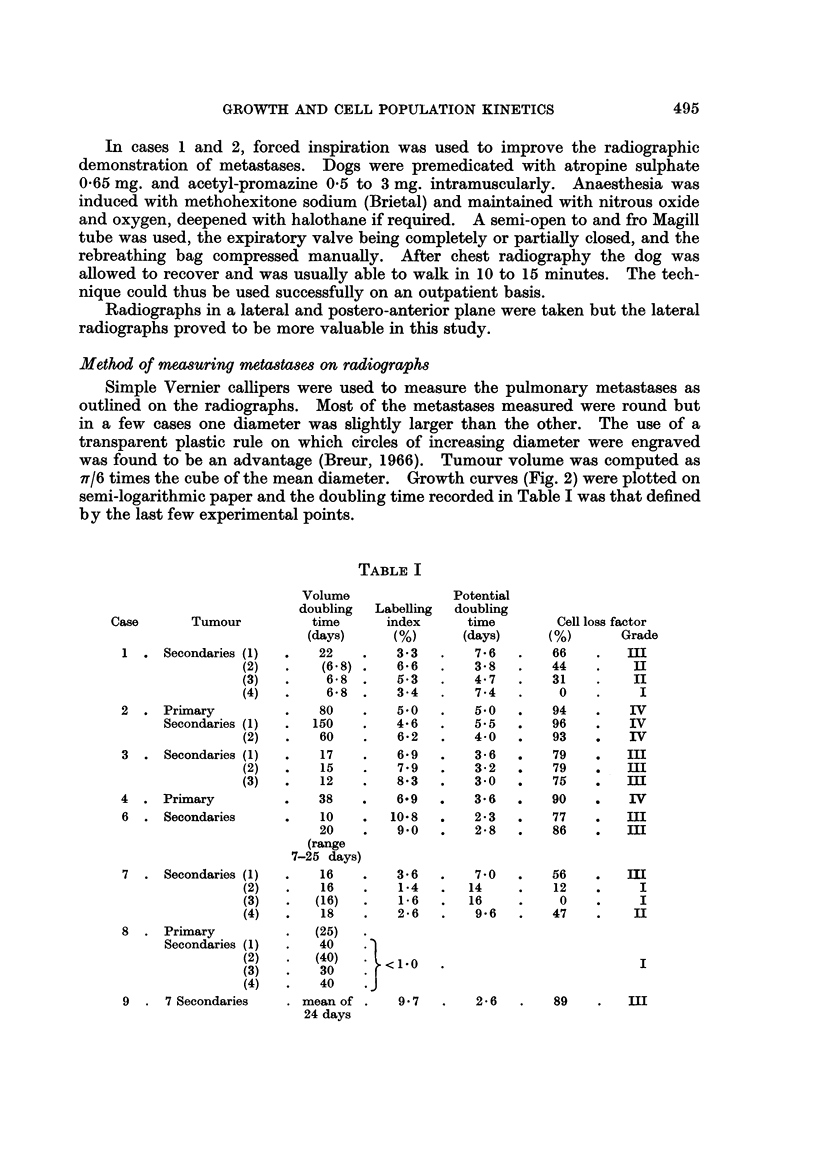

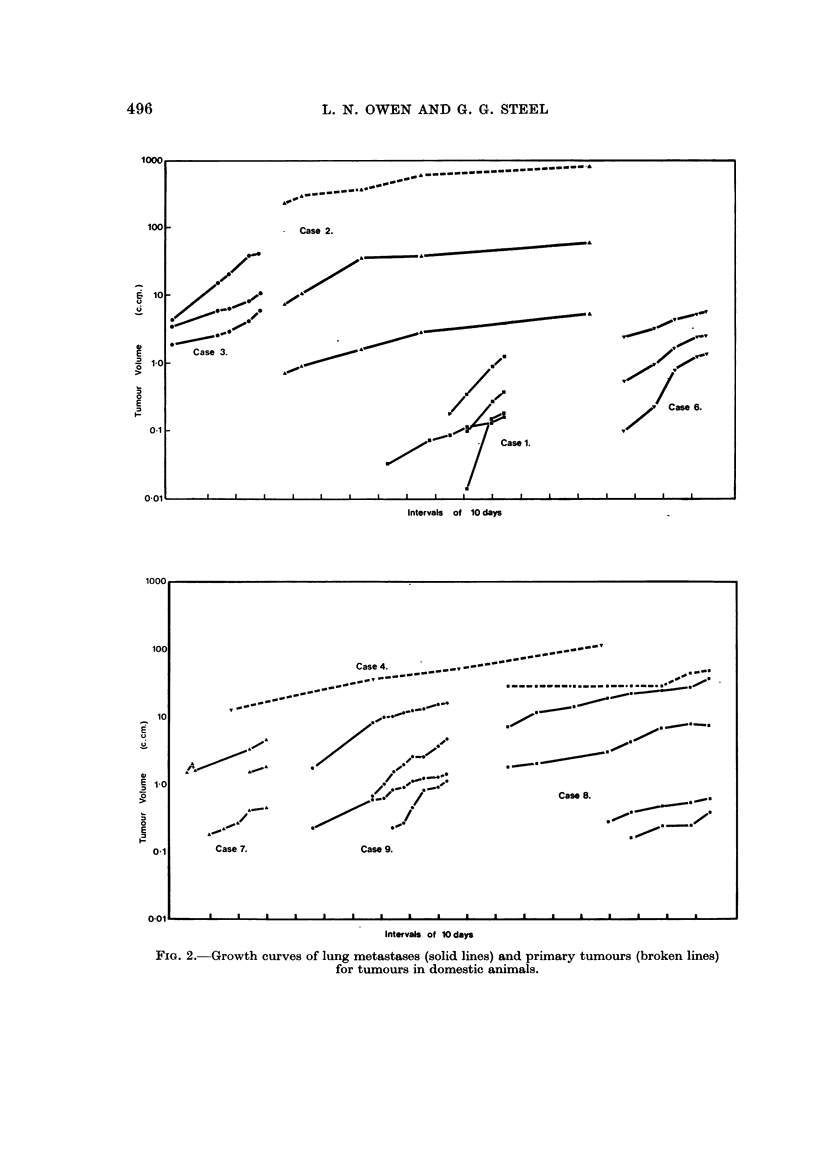

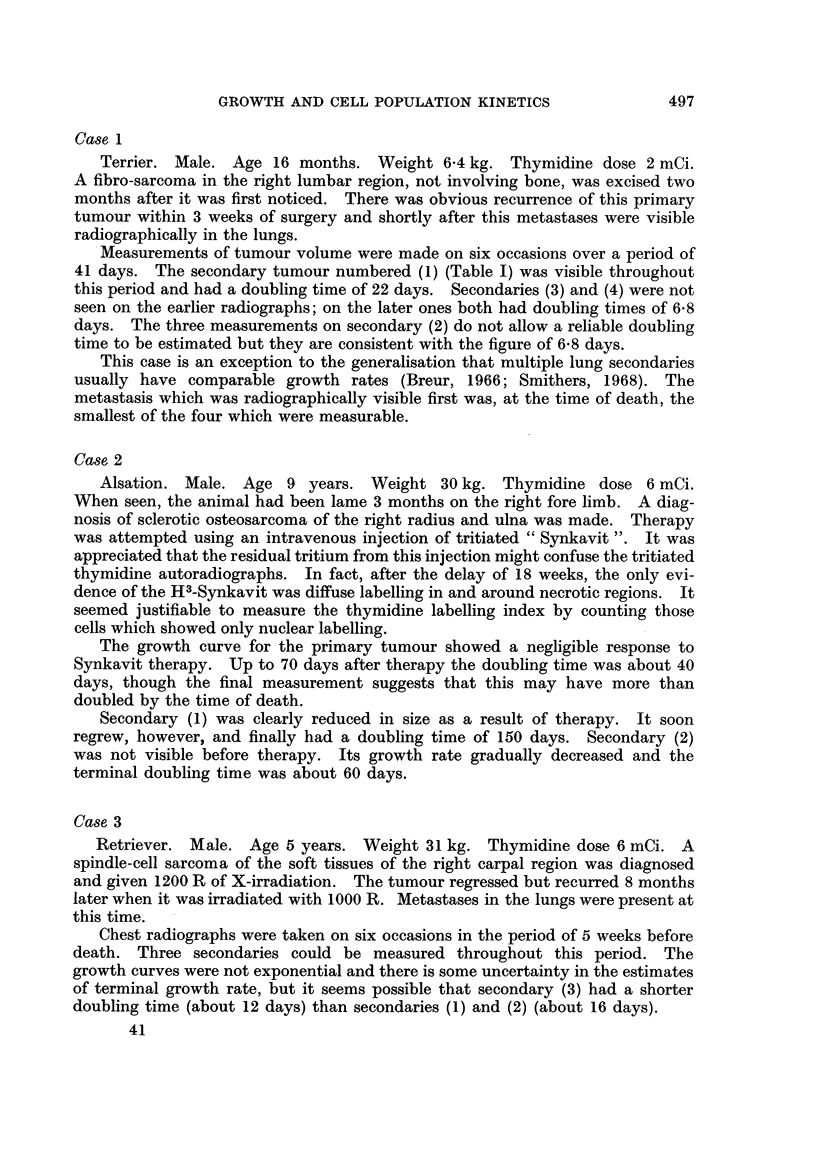

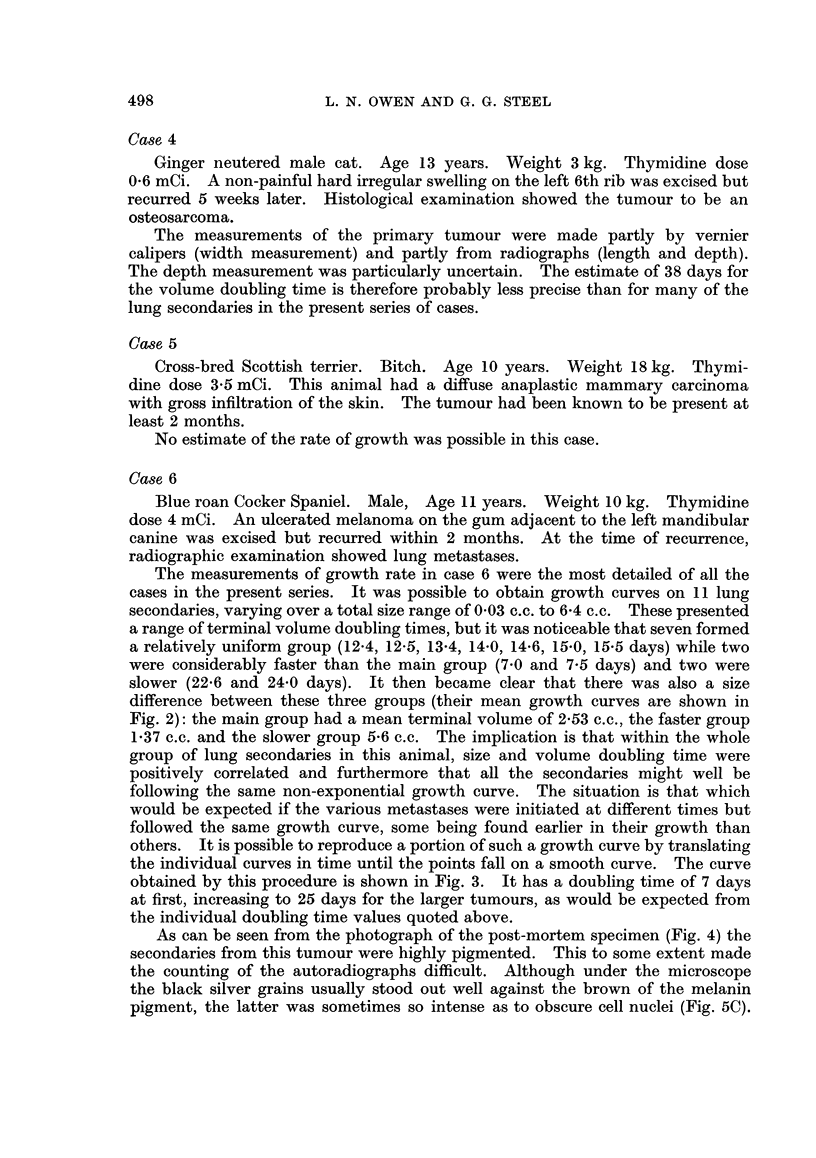

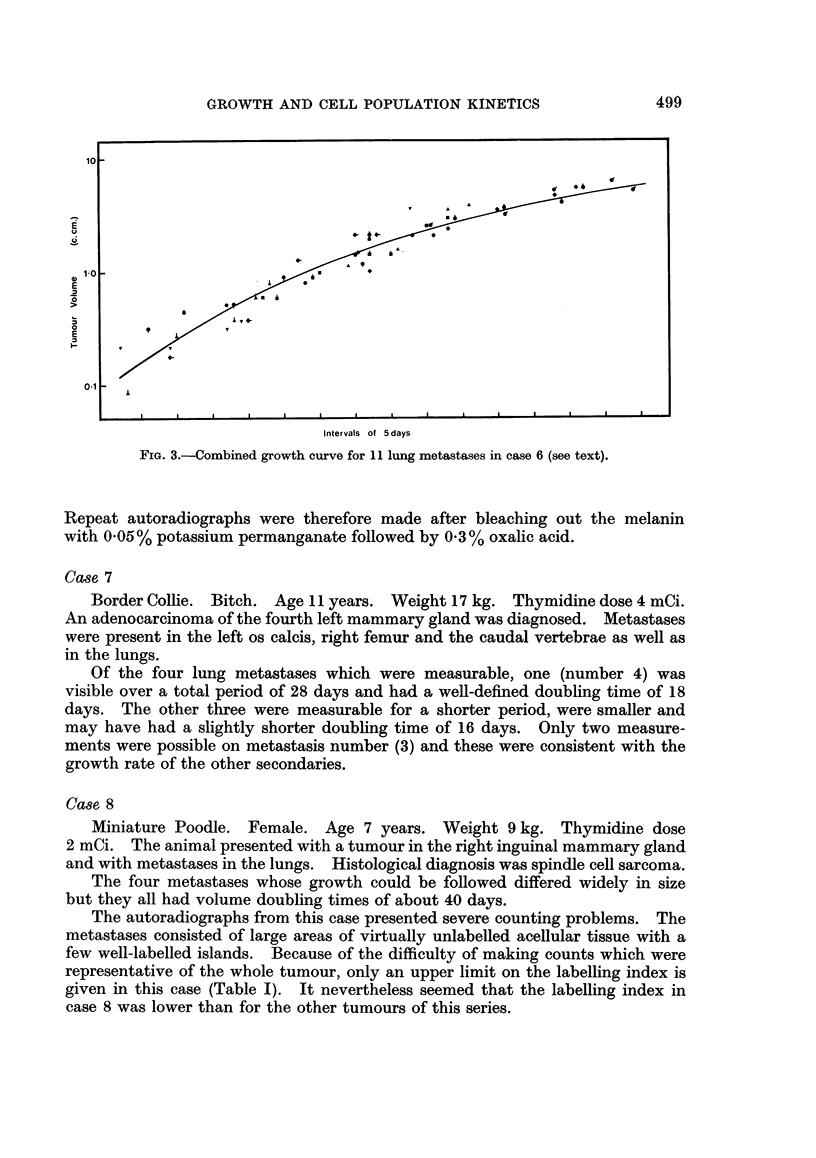

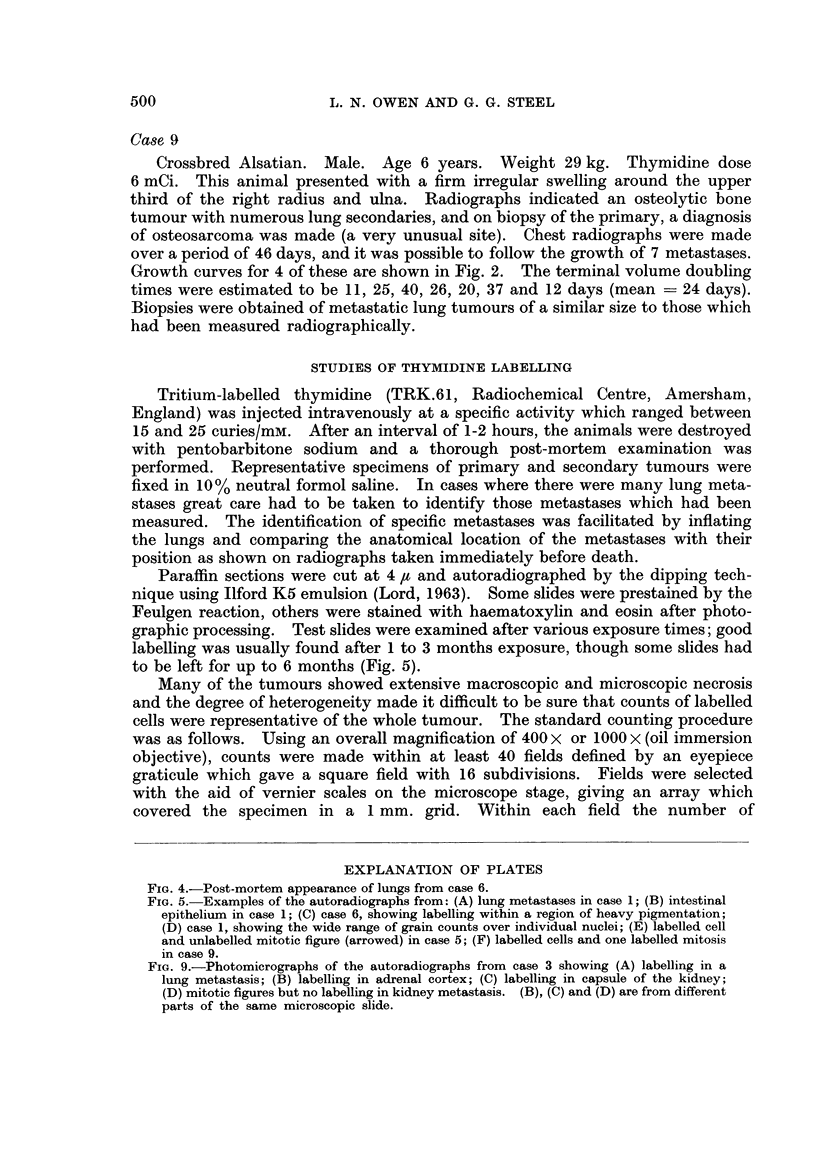

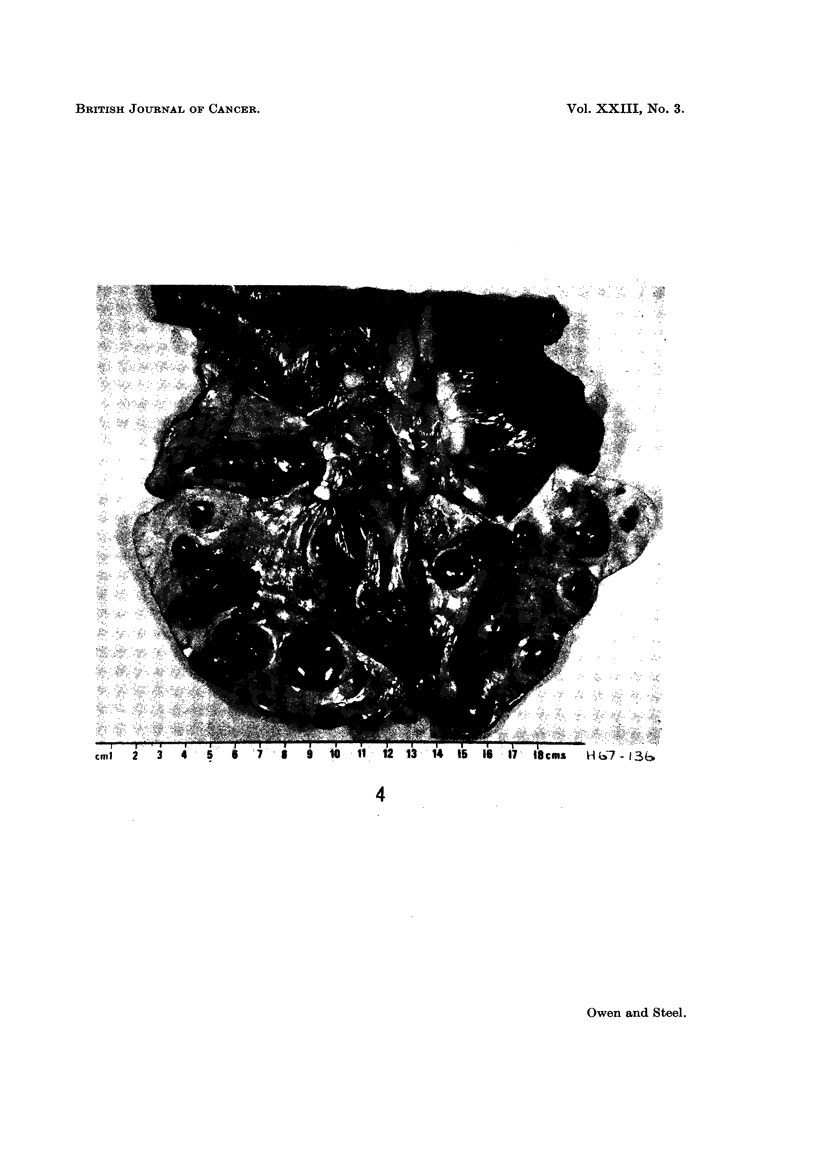

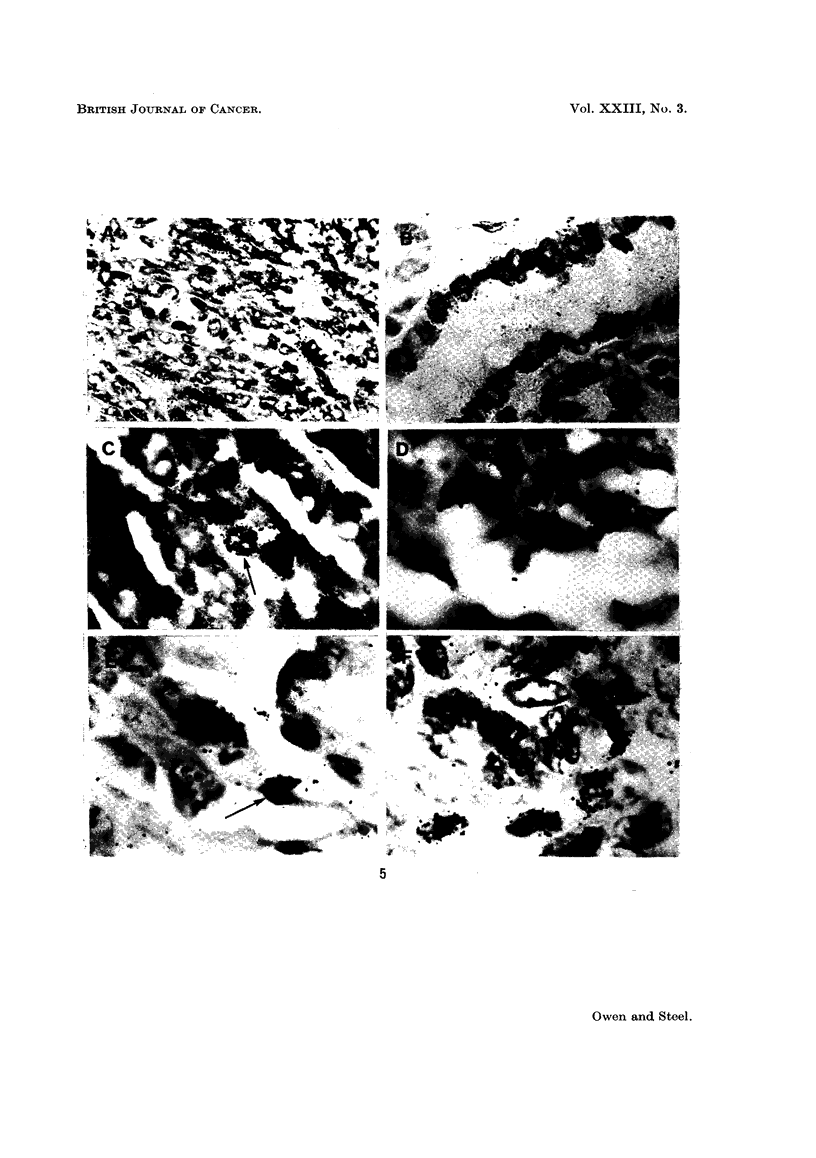

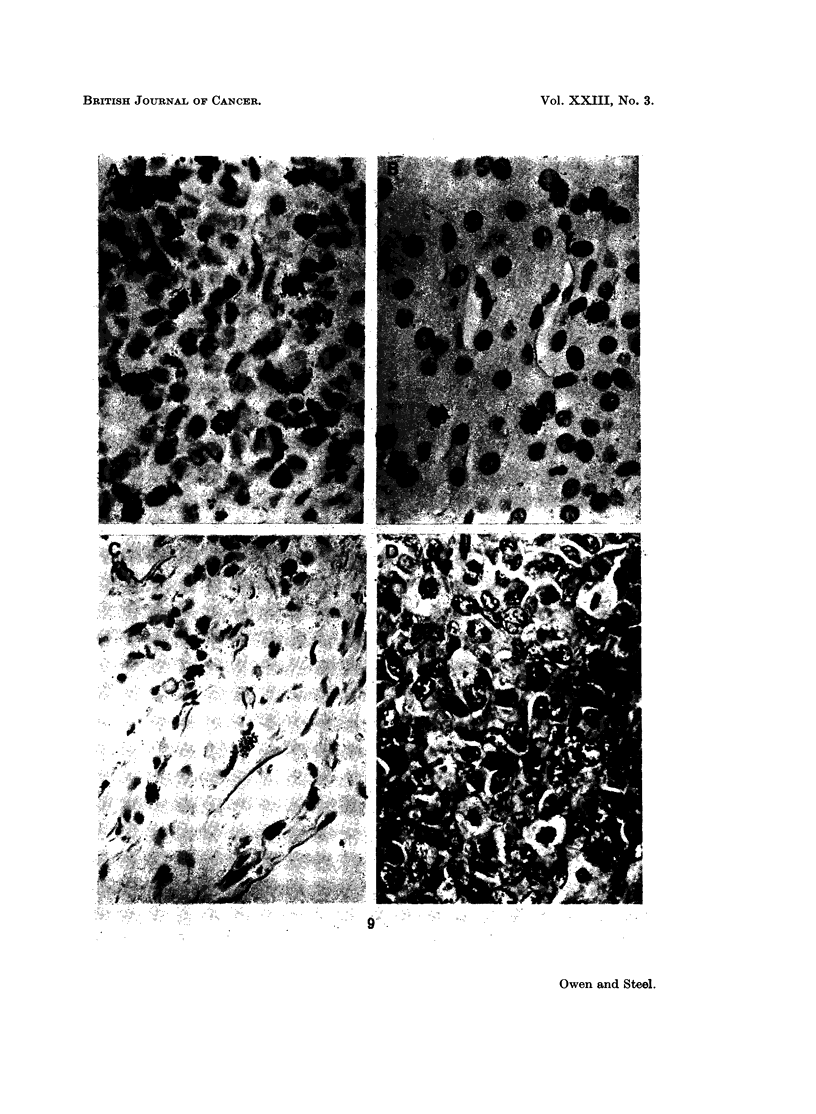

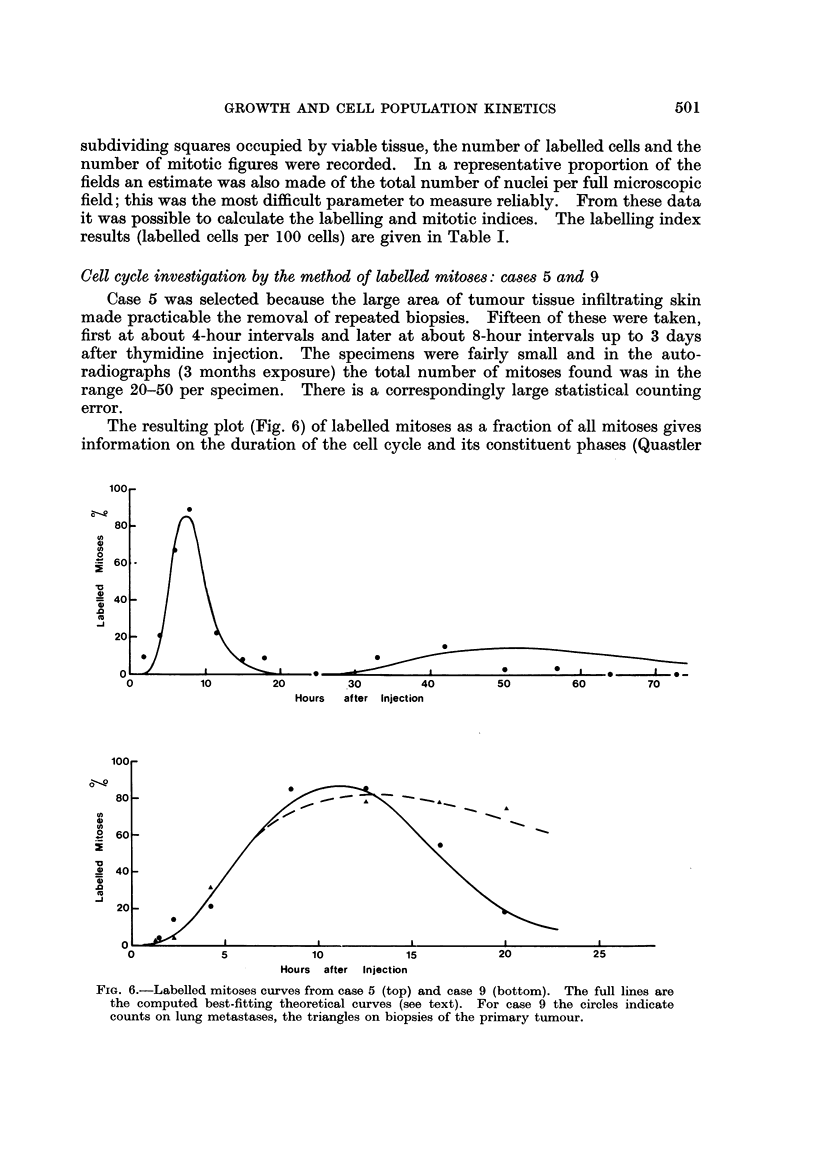

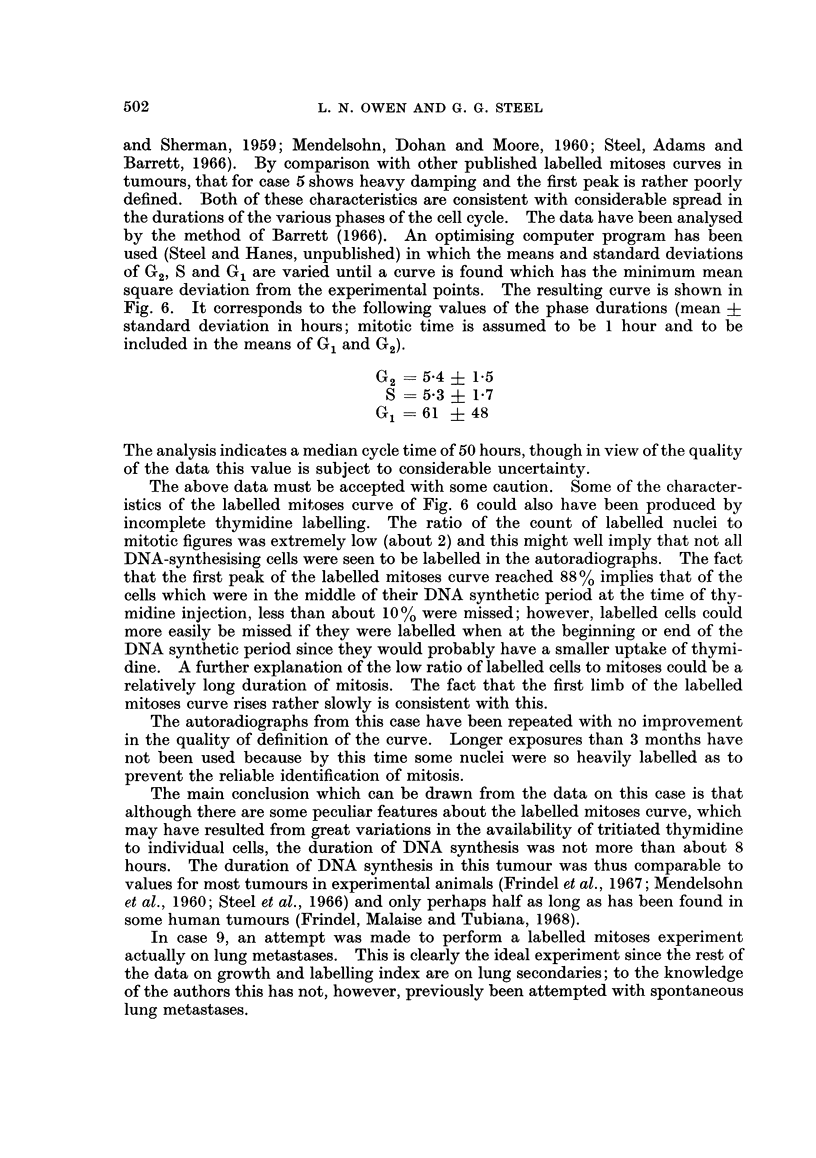

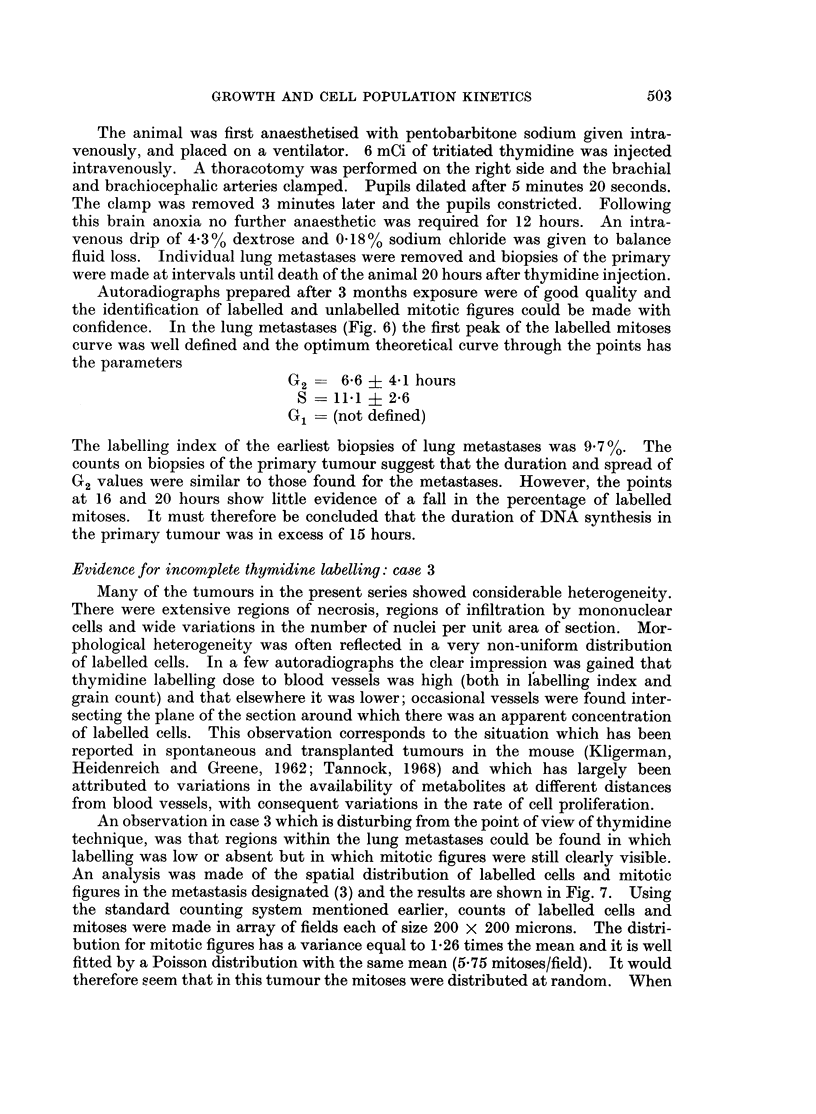

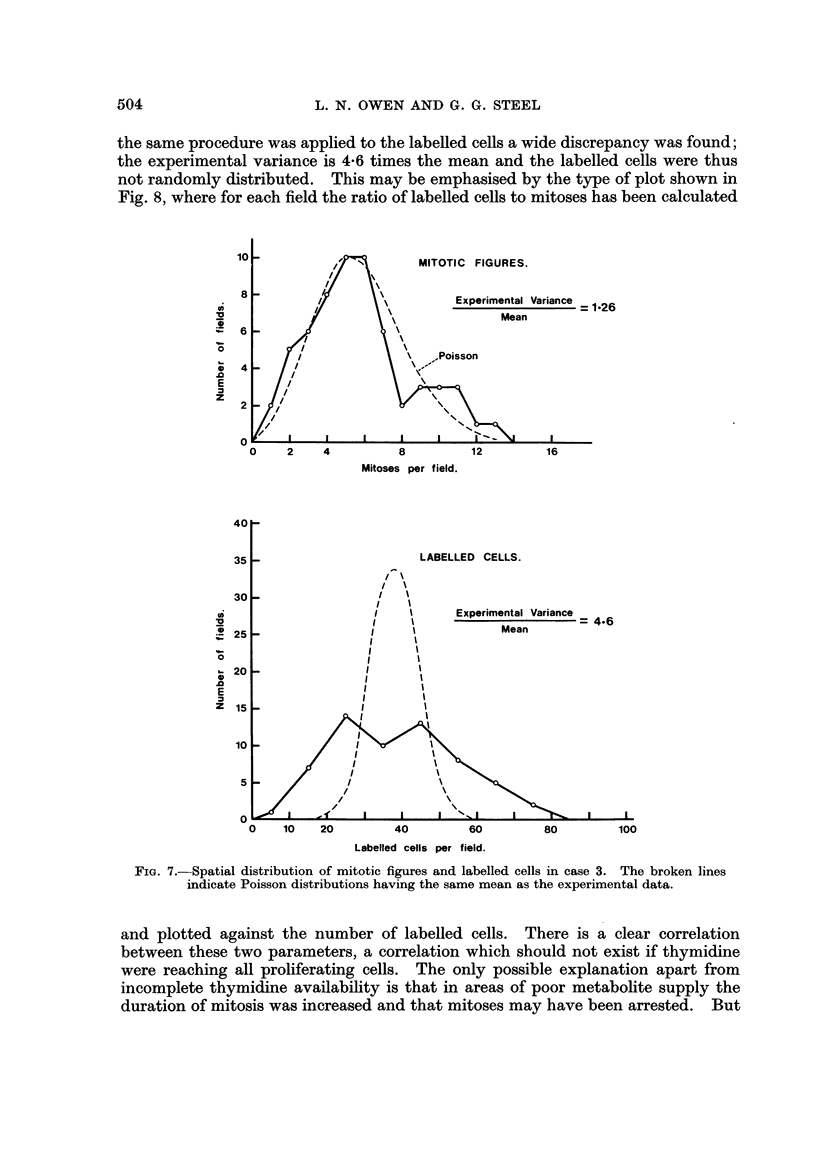

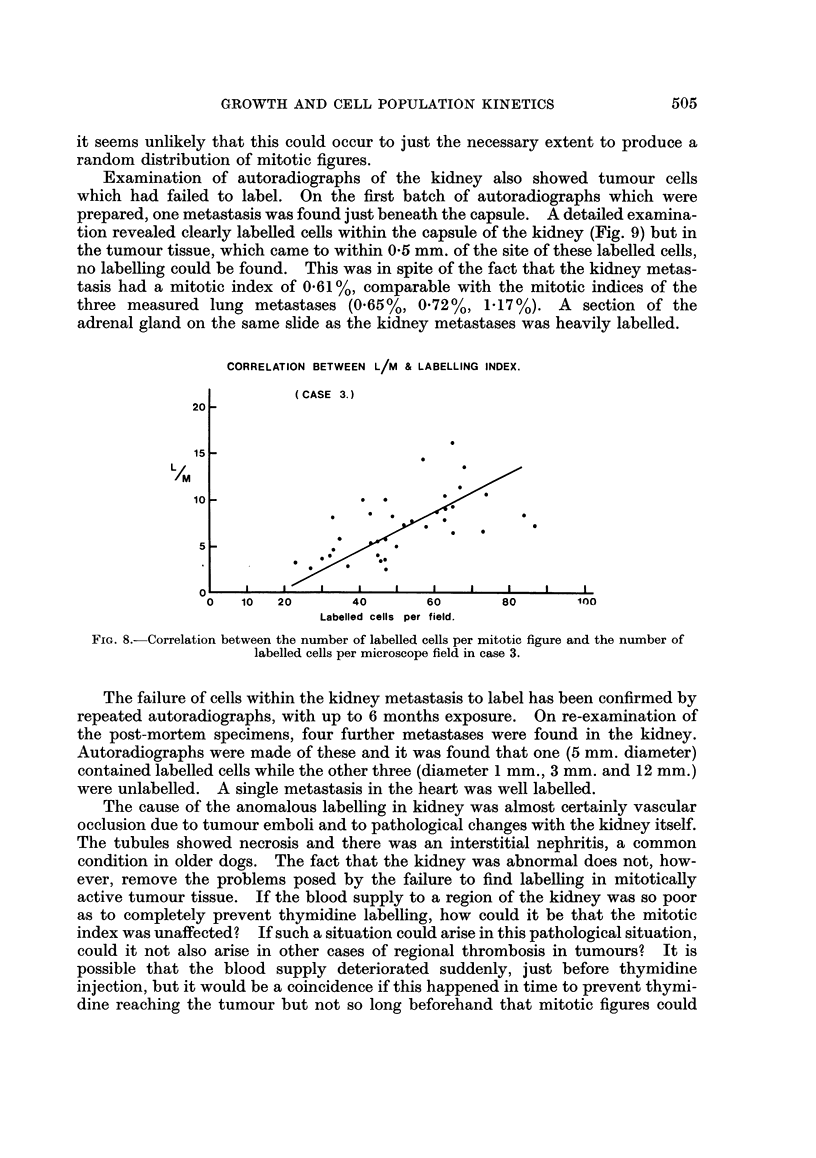

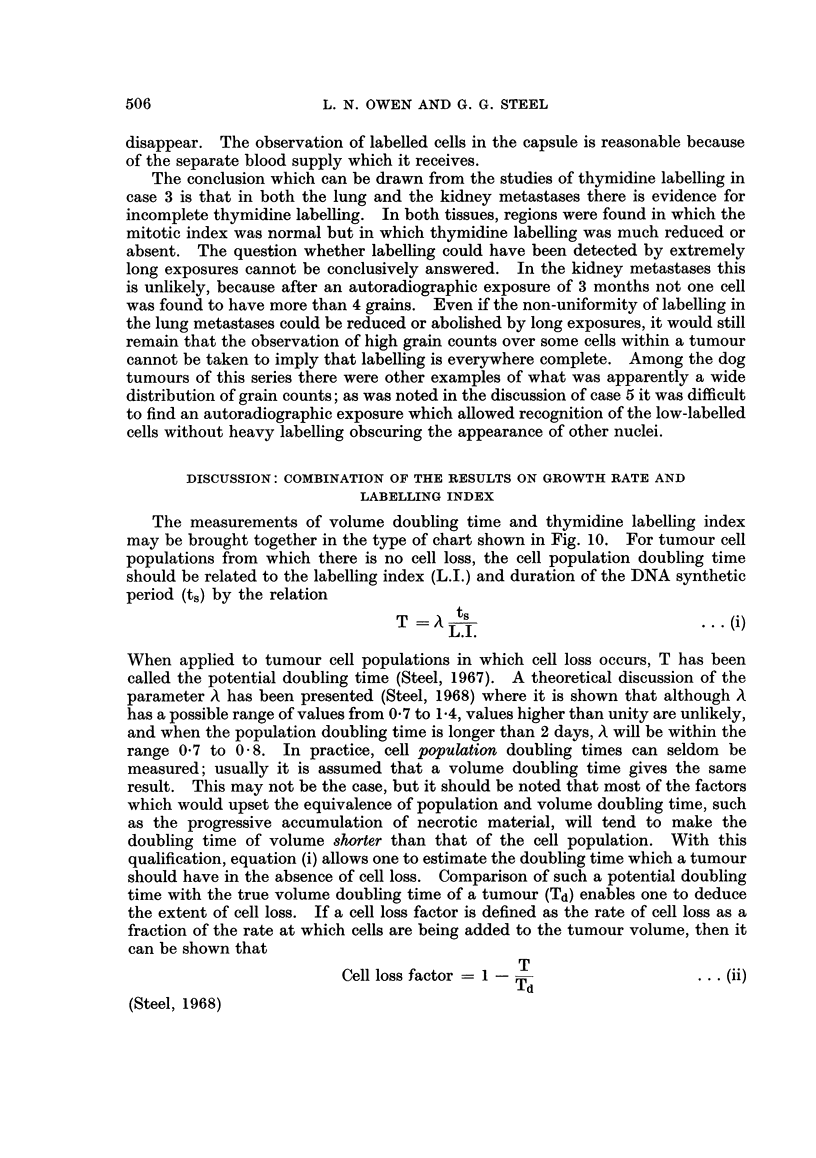

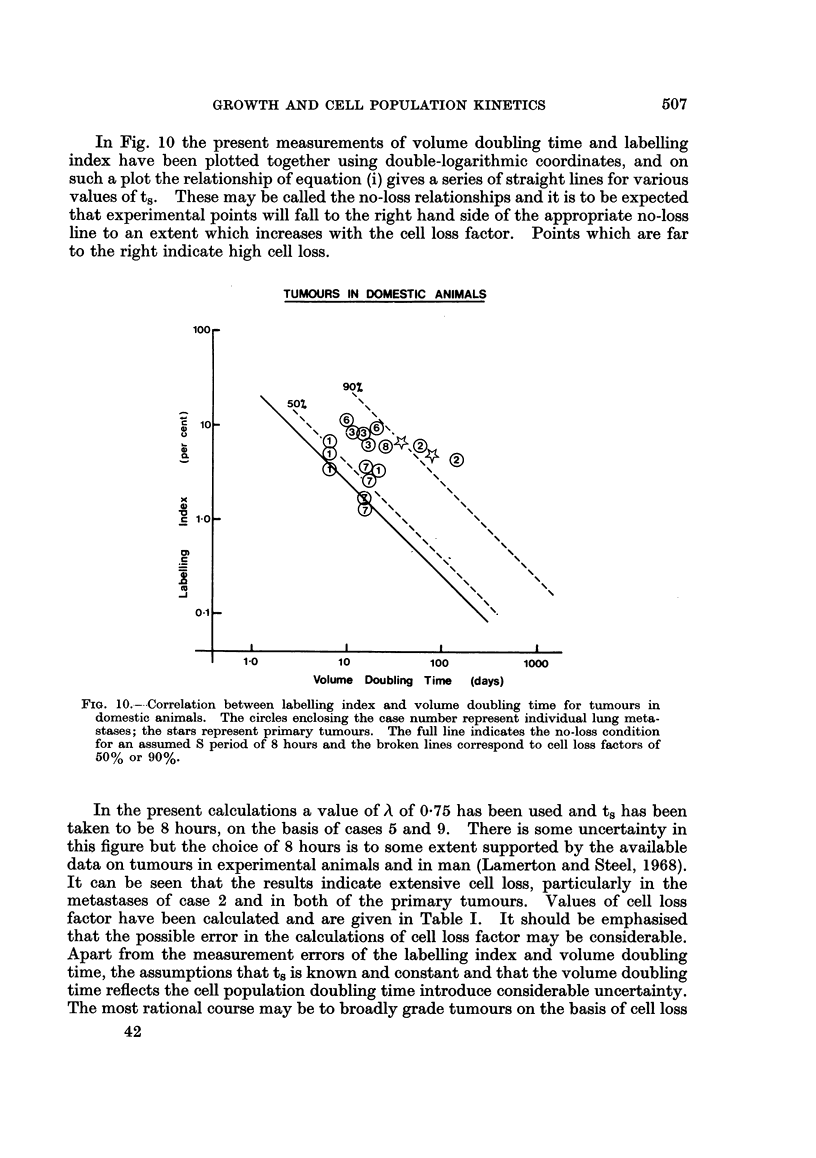

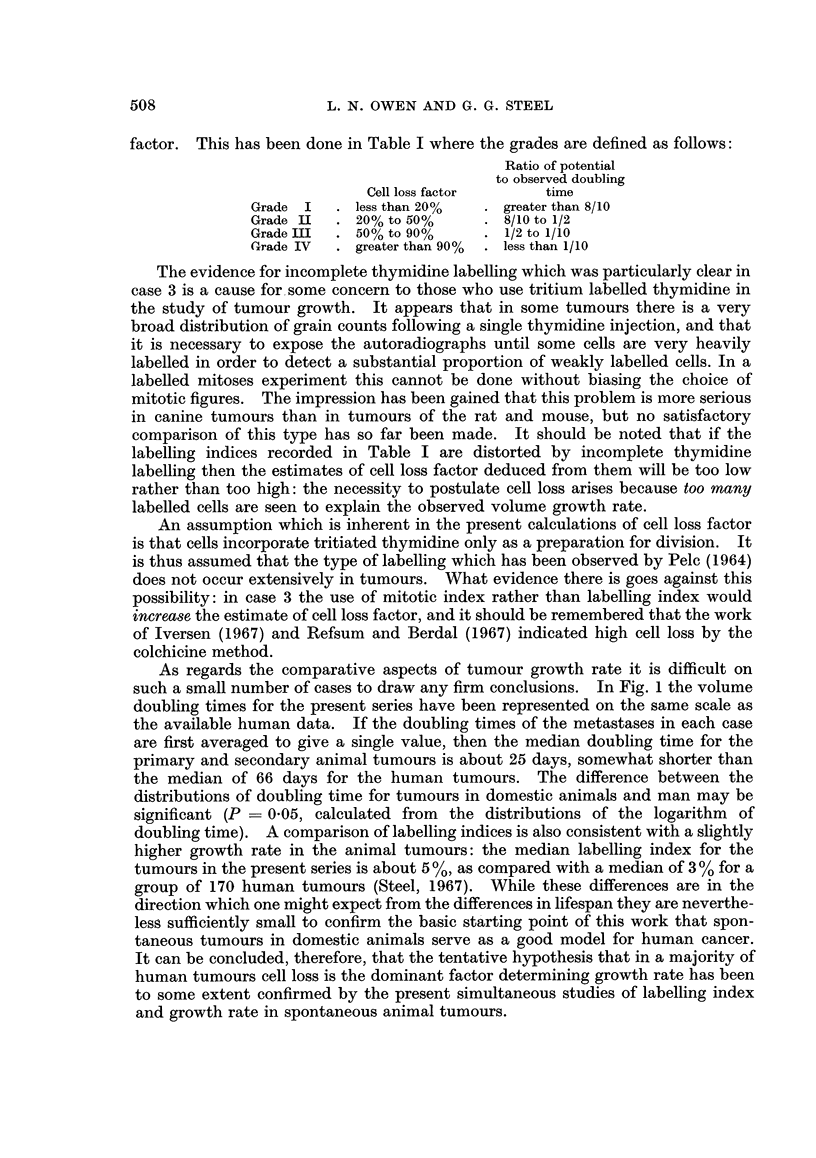

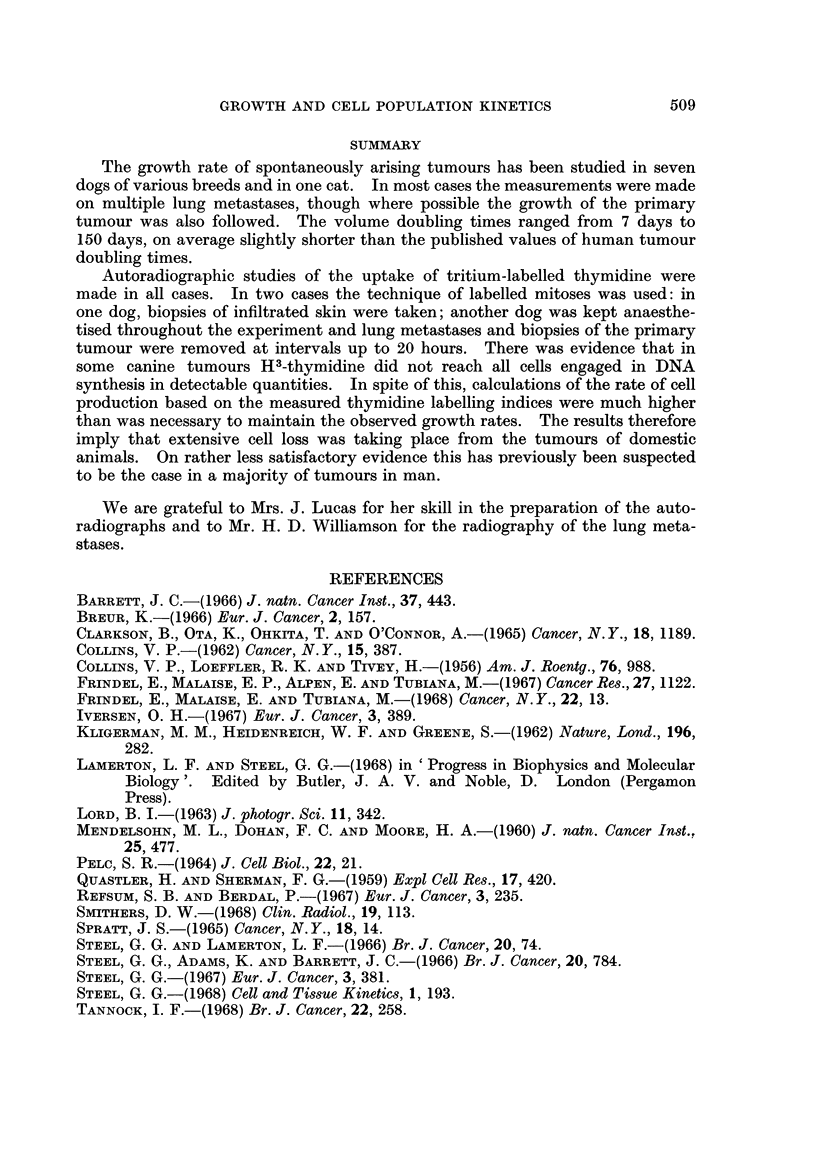


## References

[OCR_01183] Barrett J. C. (1966). A mathematical model of the mitotic cycle and its application to the interpretation of percentage labeled mitoses data.. J Natl Cancer Inst.

[OCR_01187] Clarkson B., Ota K., Ohkita T., O'Connor A. (1965). Kinetics of proliferation of cancer cells in neoplastic effusions in man.. Cancer.

[OCR_01192] Frindel E., Malaise E. P., Alpen E., Tubiana M. (1967). Kinetics of cell proliferation of an experimental tumor.. Cancer Res.

[OCR_01195] Iversen O. H. (1967). Kinetics of cellular proliferation and cell loss in human carcinomas. A discussion of methods available for in vivo studies.. Eur J Cancer.

[OCR_01206] MENDELSOHN M. L., DOHAN F. C., MOORE H. A. (1960). Autoradiographic analysis of cell proliferation in spontaneous breast cancer of C3H mouse. I. Typical cell cycle and timing of DNA synthesis.. J Natl Cancer Inst.

[OCR_01212] QUASTLER H., SHERMAN F. G. (1959). Cell population kinetics in the intestinal epithelium of the mouse.. Exp Cell Res.

[OCR_01213] Refsum S. B., Berdal P. (1967). Cell loss in malignant tumours in man.. Eur J Cancer.

[OCR_01214] Smithers D. W. (1968). Clinical assessment of growth-rate in human tumours.. Clin Radiol.

[OCR_01217] Steel G. G., Adams K., Barrett J. C. (1966). Analysis of the cell population kinetics of transplanted tumours of widely-differing growth rate.. Br J Cancer.

[OCR_01220] Steel G. G. (1967). Cell loss as a factor in the growth rate of human tumours.. Eur J Cancer.

[OCR_01223] Tannock I. F. (1968). The relation between cell proliferation and the vascular system in a transplanted mouse mammary tumour.. Br J Cancer.

